# The Case of Reactive Arthritis Secondary to* Echinococcus* Infestation

**DOI:** 10.1155/2017/3293060

**Published:** 2017-05-21

**Authors:** Bülent Alım, Sinan Çetinel, M. Alperen Servi, Fahrettin Bostancı, Mehmet Ozan Bingöl

**Affiliations:** ^1^Clinic of Physical Medicine and Rehabilitation, Bayburt State Hospital, Bayburt, Turkey; ^2^Clinic of Physical Medicine and Rehabilitation, Cumhuriyet University Hospital, Sivas, Turkey

## Abstract

Reactive arthritis is an inflammatory joint disease that develops after an infection and it usually occurs following a gastrointestinal or genitourinary system infection and it belongs to the family of arthritis called “spondyloarthritis.” We wanted to represent a rare case of reactive arthritis secondary to* Echinococcus* infestation. Cyst hydatid disease is common in endemic regions like Turkey. Internal organ involvements, especially liver and lung, are most frequent involvements. Primary bone involvement is rare complication of* Echinococcus* infestation. In our case, the patient with* Echinococcus* infection developed right knee arthritis and sacroiliitis. Other reactive and oligoarthritis causes were excluded and diagnosis of reactive arthritis secondary to cyst hydatid infestation was done with the present findings. Cold pack and TENS treatment were applied as symptomatic treatment to the right knee of the patient. Acemetacin was given as medical treatment. On the 5th day of treatment, right knee and ankle arthritis were clinically regressed. In regions where the disease is seen as endemic, such as Turkey, patients with musculoskeletal symptoms should consider the possibility of musculoskeletal involvement due to the hydatid cyst.

## 1. Introduction

Reactive arthritis is usually described as nonseptic arthritis arising in the joint after a bacterial infection [1]. It usually occurs following a gastrointestinal or genitourinary system infection and it belongs to the family of arthritis called “spondyloarthritis.” Cases of reactive arthritis that develops after many infections have been reported, although it often follows after classical agents like* Salmonella*,* Shigella*,* Campylobacter*, and* Yersinia* infections [2]. Reactive arthritis cases due to different parasitic infestations have also been reported. Most of these cases develop due to the infestation of* Giardia lamblia* parasites [3–8]. Although it is known that parasitic infestations lead to development of arthritis, there is little information in the literature on this topic [8]. Cyst hydatid disease is a parasitic infection that is seen all over the world and it is endemic in the southern parts of Middle East, Africa, South America, New Zealand, Australia, Turkey, India, and southern parts of Europe [9]. The disease most commonly occurs in liver (55–70%) and lungs (18–35%) resulting from infestation of the parasite named* Echinococcus granulosus*. More rarely, other organs and systemic involvement can also be seen. Bone involvement is around 1% [9] and it has also been reported that this disease rarely causes arthritic diseases [10]. We also wanted to represent a rare case of reactive arthritis secondary to* Echinococcus* infestation.

## 2. Case Presentation

**Figure 1:**
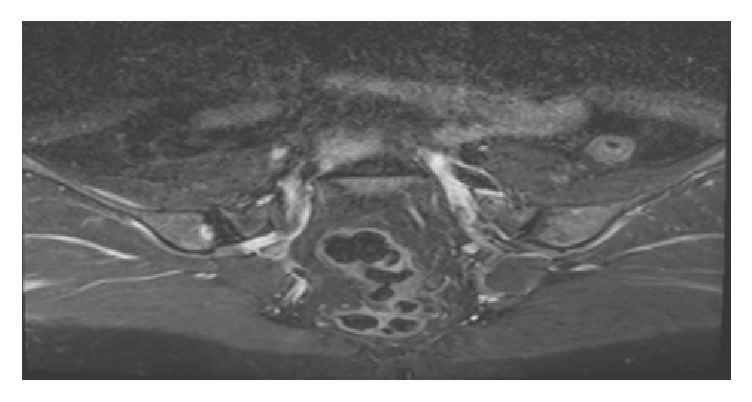
Signal enhancement consistent with the right iliac focal bone marrow edema adjacent to the right sacroiliac junction, T2 MRI sequences.

**Figure 2:**
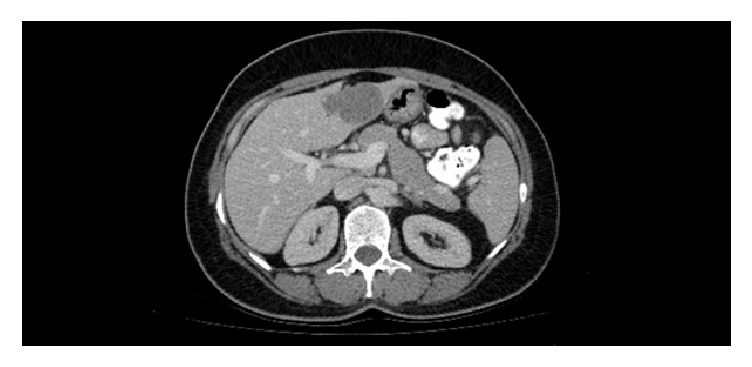
Liver hydatid cyst in abdomen tomography.

A 53-year-old female patient consulted physical medicine, rehabilitation and rheumatology department due to the pain radiating from the right inguinal region and the right hip to knee while she was being followed by general surgery clinic because of solid mass in the liver. During the assessment, patient complained of having an intermittent backache for a long time; she described a constant pain in her right hip and groin spreading to right knee that started 1 week ago and was increasing with rest. Patient also reported there was pain and slight swelling in the right ankle 10 days before that lasted for 3 days. In her background no previous illness is noted. Patient had an abdominal pain lasting for the last 6 months and abdominal ultrasound was performed; a solid mass was detected in the liver. We learned that the patient had no history of arthritis, gastroenteritis, urinary tract infection, psoriasis, or previous operation. She has not been using any medication except for analgesic. The patient had no relatives with history of inflammatory disease or malignancy in her family history. In the physical examination loss of lumbar lordosis was detected. Waist movement was slightly restricted to all directions due to pain, hand fingertip-to-floor distance was 10 cm, sacroiliac compression test was positive on the right, FABERE test was positive on the right, her right knee was warm and tender, there was no rash on the skin, local sensitivity was detected in the right upper quadrant with palpation, and other system evaluations were normal. Sacroiliac MRI was seen because of the suspicion of sacroiliitis. The sacroiliac MRI, which was assessed independently by radiology department, showed a signal enhancement consistent with the right iliac focal bone marrow edema adjacent to the right sacroiliac junction (Figure 1) and there were 2 cystic lesions at iliac front adjacent to the right hip joint. Abdomen tomography of our patient, which was requested by general surgery department, showed a well-defined 55 × 48 mm hypodense lesion with a superior calcification (lily symptom) in segment 3 (Figure 2). Hydatid cyst hemagglutination titer test was 1/320 and the patient was diagnosed as hydatid cyst disease. The patient was taken to our clinic because of development of right ankle arthritis and right knee arthritis.

In our clinic, the laboratory tests required for sacroiliitis and arthritis etiology were done. The results of these tests showed that RF was negative, anti-CCP was negative, anti-cardiolipin antibodies were negative, ds DNA was negative, ANA was negative, c-ANCA and p-ANCA were negative, sedimentation was 38 m/h, CRP was 23 mg/L, WBC was 7,31 (103/12.7 g/dL), PLT was 364 (103/*μ*L), tumor markers were negative. In addition,* Brucella* Wright and Coombs agglutination tests were negative. EBV VCA Ig M, EBV VCA Ig G, and Anti-CMV Ig G were detected as positive; EBV EA, Anti-CMV IG m were detected as negative. The right knee joint was punctured and 60 cc yellow clear liquid aspirated. At the examination of knee joint fluid Tbc DNA, tbc real time PCR, mycobacterial culture were detected as negative and there was no reproduction in cell culture; 8000 leucocytes (30% MNL, 70% PMNL) and 20 erythrocytes were detected in the cell analysis of aspiration fluid. Microscopic examination of the joint fluid showed no protoscolex and hook structures of the* Echinococcus* parasite. There was no reproduction in the blood culture. Anti-HCV, HBsAg, and Anti-HIV tests were negative. There was no evidence in the ECO for infective endocarditis. The patient consulted pulmonary medicine department. In the assessment PPD test was 5 mm and sputum culture and microscopic examination showed no Tbc* Bacillus*. HLA-B27 genetic assay was negative for spondyloarthropathy. The patient is diagnosed as reactive arthritis secondary to echinococcal infestation after all tests and no history of other diseases which can cause sacroiliitis and peripheral arthritis like SPA (spondyloarthropathy), familial Mediterranean fever, Behçet's disease, sarcoidosis, inflammatory bowel disease, and malignancy. Symptomatic treatment as 4 × 1 cold pack and TENS treatment were applied to the right knee of the patient. Acemetacin 60 mg capsule 2 × 1 was given as medical treatment. On the 5th day of treatment, right knee and ankle arthritis were clinically regressed and then patient was directed to the general surgery department for surgical treatment of hydatid cysts.

## 3. Discussion

Cyst hydatid disease is* Echinococcus granulosus* cestode larval phase infestation in tissues and it is a zoonotic parasitic disease that constitutes an important health problem in endemic regions like Turkey [11, 12]. Internal organ involvements, especially the liver and lung, are common. Primary bone involvement is rare but the incidence in the literature is 1–2.4%. Cases of femur, pelvic bone, humerus, and vertebrae involvement have been reported [13–15]. Hydatid cyst cases with bone involvement have also been reported in our country [16, 17]. In our case, 2 cysts were detected at iliac area adjacent to right sacroiliac joint and right hip joint.

In literature review, Küçükşen et al. reported that pelvic involvement of cyst hydatid disease may be confused with sacroiliitis. At the case of Küçükşen et al., patient was diagnosed with ankylosing spondylitis and followed up for 2 years. When the response was not obtained from the treatments, it was learned that patient had cystectomy operation due to hydatid disease in detailed anamnesis [18]. In our case, because of the interpretation of the lesion which was detected in the sacroiliac joint as bone marrow edema, sacroiliitis was considered in the first stage. However, as a result of further investigations, bone involvement of cyst hydatid disease was considered on detection of cystic lesions in other parts of the iliac wing. Clinical symptoms in hydatid cyst depend on depth of location and size of the cyst. The diagnosis must be with anamnesis, examination, and laboratory and radiological examinations. Lesion can be detected by ultrasonography and the diagnosis of cyst hydatid is confirmed by the appearance of the wheel pattern and the water-lily sign on the computed tomography. In our patient, abdominal ultrasonography was requested for abdominal pain and showed solid mass. Then computed tomography confirmed the diagnosis of hydatid cyst with the appearance of the wheel pattern and water-lily sign [19]. Abdomen tomography of our patient showed a well-defined 55 × 48 mm hypodense lesion with a superior calcification (lily symptom) in segment 3. In addition, various serological tests such as ELISA, immunoelectrophoresis, indirect hemagglutination, and latex agglutination tests can be used to evaluate the diagnosis, follow-up, and posttreatment relapse of the disease [20]. The indirect hemagglutination test in our patient was found positive at 1/320 titer.

The basic approach of hydatid cyst treatment is removing the cysts by open or laparoscopic operation methods and applying albendazole and praziquantel before and after operation. In addition, percutaneous approaches such as cyst puncture, aspiration, and hypertonic saline and alcohol injection to the cyst with chemotherapeutics and reaspiration are another treatment method. In some cases, albendazole monotherapy is used as a treatment modality [20–22]. In our case, for the development of arthritis in ankle and knee, surgical treatment was delayed and the patient was transferred to our clinic. Reactive arthritis and sacroiliitis were diagnosed and other etiological reasons of reactive and oligoarthritis were excluded. Diagnosis of reactive arthritis secondary to hydatid infestation was diagnosed with the present findings.

In the literature review, we did not find any case of reactive arthritis due to cyst hydatid. However, in a case reported by Sanchez Ibarrola et al., a temporal relationship between arthritic disease and cyst hydatid disease has been demonstrated. In this study, autoantibodies against cyst hydatid antigens in synovial fluid specimens were demonstrated by ELISA and immunofluorescence assessment of synovial tissue specimens showed vascular changes due to complement accumulation [10]. Protoscolex and hook structures of* Echinococcus* parasite were not observed in painted and unpainted microscopic examination of our patient's knee joint aspiration fluid. However, aspiration fluid was not examined by ELISA method and immunofluorescence examination could not be performed because synovial tissue biopsy could not be taken. The patient's clinical complaints regressed with symptomatic treatment and the patient was redirected to general surgery for the planning of surgical treatment.

## 4. Conclusion

Hydatid cyst is a parasitic disease with diffuse internal organ involvement but musculoskeletal involvement is rare. However, in regions where the disease is seen as endemic, such as Turkey, patients with musculoskeletal symptoms should consider the possibility of musculoskeletal involvement due to the hydatid cyst. Early diagnosis can prevent the secondary problems that emerged with progress of the disease and prevent unnecessary health spending. For example, Abdelhakim et al. reported that the neural pressure due to the formation of the hydatid cystic bone erosions and giant dimensions in the sacral region caused a serious problem such as cauda equina syndrome at patient and radical surgery had to be performed in the treatment of this patient [23]. In the literature, many cases have been reported where no correct diagnosis was made at an early stage and due to incomplete or incorrect diagnosis, many cases were reported with more radical treatment methods applied when the diagnosis was made [24, 25]. Therefore, detailed anamnesis, detailed physical examination, imaging methods, and serological tests in areas where the disease is endemic will help us to diagnose cyst hydatid.
